# Syne2b/Nesprin-2 Is Required for Actin Organization and Epithelial Integrity During Epiboly Movement in Zebrafish

**DOI:** 10.3389/fcell.2021.671887

**Published:** 2021-06-17

**Authors:** Yu-Long Li, Xiao-Ning Cheng, Tong Lu, Ming Shao, De-Li Shi

**Affiliations:** ^1^School of Life Sciences, Shandong University, Qingdao, China; ^2^Central People’s Hospital of Zhanjiang, Zhanjiang, China; ^3^Affiliated Hospital of Guangdong Medical University, Zhanjiang, China; ^4^Laboratory of Developmental Biology, CNRS-UMR 7622, Institut de Biologie Paris-Seine (IBPS), Sorbonne University, Paris, France

**Keywords:** Syne2b, nesprin, zebrafish, epiboly, morphogenetic movement, actin cytoskeleton, epithelial integrity

## Abstract

Syne2b/nesprin-2 is a giant protein implicated in tethering the nucleus to the cytoskeleton and plays an important role in maintaining cellular architecture. Epiboly is a conserved morphogenetic movement that involves extensive spreading and thinning of the epithelial blastoderm to shape the embryo and organize the three germ layers. Dynamic cytoskeletal organization is critical for this process, but how it is regulated remains elusive. Here we generated a zebrafish *syne2b* mutant line and analyzed the effects of impaired Syne2b function during early development. By CRISPR/Cas9-mediated genome editing, we obtained a large deletion in the *syne2b* locus, predicted to cause truncation of the nuclear localization KASH domain in the translated protein. Maternal and zygotic *syne2b* embryos showed delayed epiboly initiation and progression without defects in embryonic patterning. Remarkably, disruption of Syne2b function severely impaired cytoskeletal organization across the embryo, leading to aberrant clustering of F-actin at multiple cell contact regions and abnormal cell shape changes. These caused disintegration of the epithelial blastoderm before the end of gastrulation in most severely affected embryos. Moreover, the migration of yolk nuclear syncytium also became defective, likely due to disorganized cytoskeletal networks at the blastoderm margin and in the yolk cell. These findings demonstrate an essential function of Syne2b in maintaining cytoskeletal architecture and epithelial integrity during epiboly movement.

## Introduction

Nesprins (Nuclear Envelope SPectrin Repeat proteINS) are outer nuclear membrane resident macromolecules that constitute the LINC (LInker of the Nucleoskeleton and Cytoskeleton) complex (Zhang et al., 2001; Rajgor and Shanahan, 2013; Davidson and Cadot, 2020). Nesprin-1 and –2 are giant proteins containing an N-terminal actin-binding domain, followed by a long central domain composed of spectrin repeats, and a C-terminal nuclear localization KASH (Klarsicht/ANC-1/Syne Homology) domain, thus connecting the nucleus to the cytoplasmic actin cytoskeleton (Crisp et al., 2006; Noegel and Neumann, 2011). In humans, these proteins are encoded by *SYNE* (*synaptic nuclear envelope*) *–1* and *–2* loci, whose mutations cause various diseases, such as muscular dystrophy, dilated cardiomyopathy, neurological disorders and hearing loss (Cartwright and Karakesisoglou, 2014; Janin and Gache, 2018; Zhou et al., 2018a). Mutant mouse models for nesprins that mimic different human diseases have provided insights into their tissue-specific postnatal functions (Zhou et al., 2018b). However, the implication of nesprins in morphogenetic movements remains unclear.

Epiboly is a conserved morphogenetic process in vertebrates (Solnica-Krezel, 2005). In the zebrafish embryo, it is initiated in the blastula and progresses in the gastrula. By the mid-blastula stage, the embryo becomes organized into a superficial monolayer known as the enveloping layer (EVL), a deep cell multilayer (DEL), and a yolk syncytial layer (YSL) consisting of nuclei (YSN) and non-yolky cytoplasm located on the yolk cell (Kimmel et al., 1995). Epiboly initiates when the large yolk cell domes upward into the blastoderm. During epiboly progression, the blastoderm and YSL move toward the vegetal pole to engulf the yolk cell by the end of gastrulation (Warga and Kimmel, 1990; Bruce, 2016). Dynamic cytoskeletal changes across the embryo are important for epiboly movement. Cortical F-actin belt is organized in EVL cells from the early blastula stage onward; punctate F-actin rings form ahead of the leading edge of the E-YSL at more late stages of epiboly; F-actin bundles are also present in the vegetal cortex of the yolk cell until before the end of gastrulation (Lee, 2014). These actin networks play essential roles in modulating cellular behavior changes and cell rearrangements during epiboly progression (Köppen et al., 2006; Li et al., 2017; Sun et al., 2017). Thus, cytoskeletal organization is closely linked to epithelial morphogenesis, but how it is regulated remains largely elusive.

Here we report a role for Syne2b (also called nesprin-2) during epiboly in zebrafish. By CRISPR/Cas9-mediated genome editing, we generated a deletion mutation in the zebrafish *syne2b* locus that should impair the attachment of Syne2b to the nuclear membrane. Maternal and zygotic *syne2b* mutants displayed severely disorganized actin cytoskeleton and delayed epiboly movement. Strikingly, cortical F-actin belt in the EVL was reduced and abnormally concentrated to multiple cell contact regions, resulting in aberrant cell shape changes and disrupted epithelial integrity. These results demonstrate a requirement for Syne2b in regulating cytoskeletal organization to maintain cell shape and integrity of the epithelial blastoderm. They provide insights into the implication of nesprins in morphogenetic movements during vertebrate early development.

## Methods

### Zebrafish

Adult zebrafish were maintained in standard housing systems. Embryos were microinjected using a PLI-100A picoliter microinjector (Harvard Apparatus).

### Ethics Statement

All experiments were approved by the Ethics Committee for Animal Research of Life Science of Shandong University (SYDWLL-2018–05), and performed by following the ARRIVE guidelines.

### Genome Editing of *Syne2b* Locus

DNA templates for *in vitro* transcription of sgRNAs were cloned into p-T7-gRNA vector. The two sgRNAs (200 pg each) were mixed with Cas9 protein (300 pg) and injected into 1-cell stage embryos. Fish were screened by sequencing PCR products amplified from tail fin genomic DNA as described previously (Shao et al., 2020).

### Expression Constructs and mRNA Synthesis

The sequence encoding the last 69 amino acids of zebrafish Syne1a was amplified by PCR (Supplementary Table 1) and cloned inframe with the myc-coding sequence in the pCS2 vector, to generate the dominant negative Syne1a KASH (Tsujikawa et al., 2007). Life-Act-GFP was cloned in the pCS2 vector, and mGFP and H2B-RFP were described previously (Cheng et al., 2017). Capped mRNAs were *in vitro* transcribed using mMESSAGE mMACHINE SP6 kit (Ambion).

### Phalloidin and DAPI Staining

Stage-matched wild-type and MZ*syne2b* embryos were fixed in 4% paraformaldehyde for 1 h at room temperature. They were stained with rhodamine-conjugated phalloidin and counterstained with DAPI (Sigma-Aldrich). Images were acquired using a confocal microscope (Zeiss LSM700). Z-stack projections were generated using the z-projection function.

### Time-Lapse Imaging

Embryos were mounted in a cavity microscope slide in 1% low-melting agarose. Cell shape changes in the EVL and vegetal movements of YSN were recorded for 1 h and 30 min, respectively, at 5 min intervals. Time-lapse movies were generated using ImageJ software (NIH Image). The experiments were repeated twice using 6 wild-type or MZ*syne2b* embryos from different batches.

### Immunofluorescence

Embryos were fixed in 4% paraformaldehyde and rinsed in phosphate-buffered saline. They were incubated with rabbit polyclonal antibodies against ß-tubulin (1/1,000, Sigma-Aldrich), followed by fluorescein-conjugated secondary antibody. The samples were analyzed under a confocal microscope (Zeiss, LSM700).

### qRT-PCR

Total RNA was extracted using TRIzol Reagent (Invitrogen) and reverse transcribed using M-MLV reverse transcriptase (Invitrogen) in the presence of random primer. Gene-specific primers are listed in Supplementary Table 1.

### Whole-Mount *in situ* Hybridization

DNA templates for generating *syne2b*, *goosecoid*, *chordin* and *tbxta* probes were obtained by PCR amplification of embryonic cDNAs (Supplementary Table 1). PCR products were cloned in pBluescript or pGEM-T easy vector and antisense probes were synthesized using appropriate RNA polymerases and digoxigenin-11-UTP (Roche Diagnostics). *In situ* hybridization was performed according to published protocol (Thisse and Thisse, 2008). Embryos after 24 hpf (hours post-fertilization) were treated with proteinase K (10 μg/mL) for 15 min.

### Statistical Analyses

Data from two to three independent experiments were statistically analyzed using unpaired Student’s *t*-test, with *p-*values indicated in the corresponding figures and legends.

## Results

### Expression of *Syne2b* During Early Development

**FIGURE 1:**
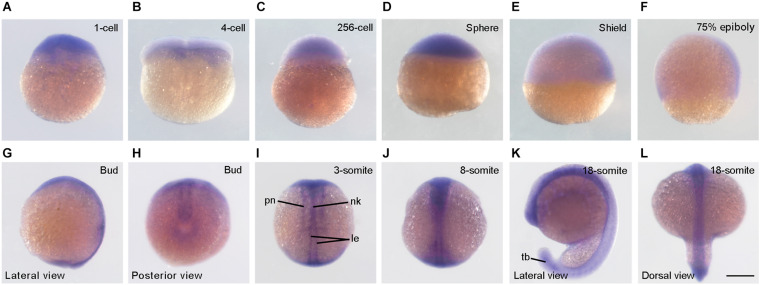
Expression pattern of *syne2b* during early development. **(A–D)** At cleavage and blastula stages, *syne2b* transcripts are enriched in the blastodisc. Animal pole region is on the top. **(E,F)** Predominant expression of *syne2b* in the dorsal region during gastrulation. Lateral view with animal pole region on the top and dorsal region to the right. **(G,H)** Expression of *syne2b* along the anteroposterior axis at bud stage. **(I,J)** Dorsal views show *syne2b* expression in axial structures, including neural keel (nk), lateral edges (le) of neural keel and primary neurons (pn). **(K,L)** Expression of *syne2b* in the neural tissue and tail bud (tb). Scale bar: **(A–L)** 200 μm.

We first performed *in situ* hybridization to analyze *syne2b* expression pattern. Maternal *syne2b* transcripts were enriched in the blastodisc, but were also weakly present in the yolk region at least at 1-cell stage (Figures 1A–D). During gastrulation, *syne2b* expression was detected in the blastoderm, particularly in the dorsal region (Figures 1E,F). As development proceeds, *syne2b* transcripts became predominantly localized to dorsal structures, such as neural keel in the anterior region, lateral edges of neural keel in the posterior region, and primary neurons (Figures 1G–J). At 18-somite stage, *syne2b* expression was also evident in the tail bud (Figures 1K,L). Thus, Syne2b may be involved in early developmental processes before and after zygotic genome activation beginning around 512-cell stage.

### Generation of *Syne2b* Deletion Mutants

**FIGURE 2:**
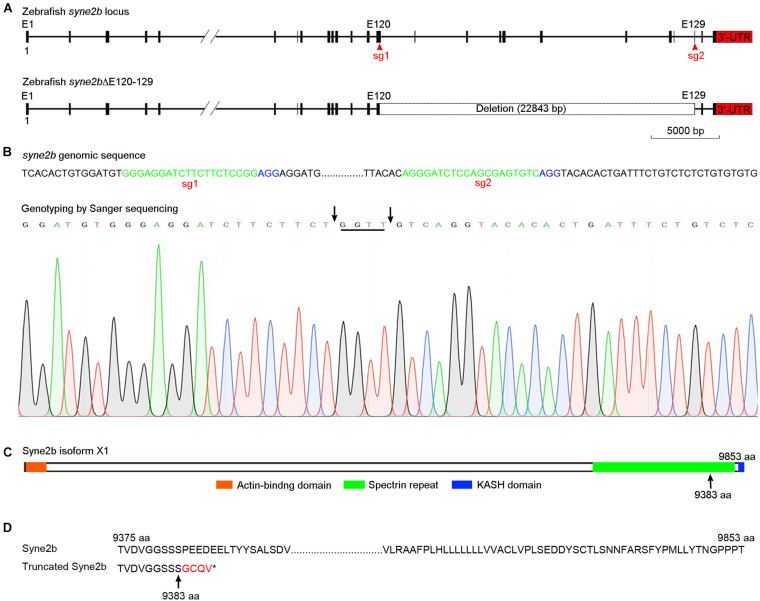
Generation of *syne2b* mutation by CRISPR/Cas9-mediated genome editing. **(A)** Organization of the zebrafish *syne2b* locus. Shown are the sgRNA targeting sites (sg1 and sg2) and the deleted intervening region. **(B)** Genotyping of *syne2b* mutation. The sgRNA recognition sequences are green colored, and the PAM regions are shown in blue. Arrows indicate the positions of deletion in exons 120 and 129. Nucleotides inserted as a result of DNA repair are underlined. **(C)** Syne2b protein domains. Arrow indicates the position after which truncation occurs in the translated Syne2b protein. **(D)** Alignment of the C-terminal regions from wild-type and truncated Syne2b proteins. Residues translated after frameshift are indicated in red.

Zebrafish *syne2b* locus (NCBI gene ID: 559348) comprises 131 exons and is predicated to encode a giant protein consisting of 9,853 amino acids. As human SYNE2/Nesprin-2, zebrafish Syne2b contains an N-terminal actin-binding domain, followed by spectrin repeats and a C-terminal KASH domain. To disrupt *syne2b* gene by CRISPR/Cas9, we synthesized two sgRNAs targeting exons 120 and 129, respectively. PCR-based genotyping of genomic DNA (Supplementary Figure 1) in F1 and F2 offspring detected a large deletion of the intervening region (Figures 2A,B). Sequencing of PCR products indicated that this caused a frameshift and the premature termination of translation after amino acid position 9383, predicting the expression of a truncated protein without attachment to the nuclear membrane due to the absence of the nuclear localization KASH domain (Figures 2C,D). Analysis by qRT-PCR using primers that are specific for each isoform of *syne* genes showed a less than twofold decreased expression of *syne2b* mutant transcripts in MZ*syne2b* embryos at 10 hpf, probably caused by nonsense-mediated decay. The expression of *syne2a* and *syne3*, but not of *syne1a* and *syne1b*, was also slightly but significantly decreased (Supplementary Figure 2). Thus, this mutation may affect the function of Syne2b protein and the expression of *syne2a* and *syne3* genes.

### Disruption of Syne2b Function Delays Epiboly Initiation and Progression

The phenotype of *syne2b* mutants was examined in comparison with time-matched wild-type embryos. To avoid possible variations in the exact timing of fertilization, which may introduce fluctuations in embryonic development, we collected eggs spawned within 10 min intervals and monitored the number of blastomeres from 8 to 32-cell stages. Heterozygous and zygotic homozygous *syne2b* mutants showed no developmental defects. Maternal-zygotic *syne2b* (MZ*syne2b*) embryos developed normally during early cleavage stages, but they were transiently higher from 128 to 512-cell stages (Supplementary Figure 3). After resuming a spherical shape at 4 hpf, the subsequent development showed delayed epiboly initiation and progression, with 100% penetrance.

**FIGURE 3:**
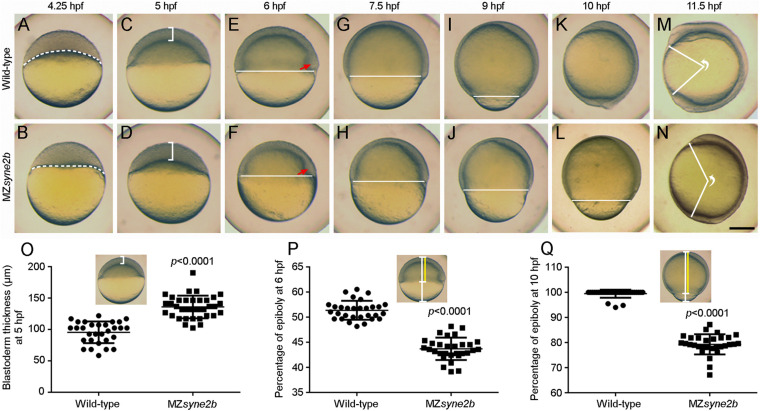
Disruption of Syne2b function impairs epiboly movement and anteroposterior axis elongation. Live images show epiboly initiation and progression in time-matched embryos. Lateral views with animal pole or anterior region on the top. **(A,B)** At 4.25 hpf, yolk cell domes into the blastoderm in wild-type embryos, while the border between the blastoderm and the yolk cell (broken lines) remains flat in MZ*syne2b* embryos. **(C,D)** At 5 hpf, the blastoderm in MZ*syne2b* embryos is thicker compared with wild-type embryos (brackets). **(E,F)** At 6 hpf, MZ*syne2b* embryos show delayed epiboly compared with wild-type embryos (horizontal lines). Red arrows indicate the embryonic shield. **(G–J)** At 7.5 and 9 hpf, delayed epiboly progression is evident in MZ*syne2b* embryos. **(K,L)** At 10 hpf, wild-type embryos complete gastrulation, whereas MZ*syne2b* embryos only reach about 80% epiboly. **(M,N)** At 11.5 hpf, wild-type embryos develop a long anteroposterior axis, while MZ*syne2b* embryos only complete gastrulation. The angle formed between the most anterior end and the most posterior end of the anteroposterior axis, with vertex at the geometric center of the embryo, reflects the extent of axis elongation. **(O)** Scatter plot compares blastoderm thickness between wild-type and MZ*syne2b* embryos. **(P,Q)** Scatter plots compare epiboly progression between wild-type and MZ*syne2b* embryos. Scale bars: **(A–N)** 200 μm.

At 4.25 hpf when epiboly initiated in wild-type embryos with the yolk cell doming into the blastoderm, there was still a flat border between the blastoderm and the yolk cell in MZ*syne2b* embryos (Figures 3A,B). At 5 hpf, the blastoderm became thinner in wild-type embryos, whereas it remained thicker in MZ*syne2b* embryos (Figures 3C,D,O), indicating delayed yolk cell doming. From 6 to 10 hpf, there was obviously a delayed epiboly progression in MZ*syne2b* embryos, as judged by the slowed spreading of the blastoderm toward the vegetal pole (Figures 3E–L,P,Q). As a result, when wild-type embryos completed gastrulation at 10 hpf (Figure 3K), MZ*syne2b* embryos only reached about 80% epiboly (Figure 3L). At 11.5 hpf, although most MZ*syne2b* embryos could complete epiboly, they presented a shortened anteroposterior axis compared to time-matched wild-type embryos (Figures 3M,N). Thus, MZ*syne2b* embryos showed a delay of about 1.5 h in epiboly progression. This is significant given that the period from the initiation to the end of epiboly normally lasts 6 h when embryos develop at 28.5°C (Kimmel et al., 1995).

We also observed a proportion of more severely affected embryos that could not complete gastrulation due to disintegration of the blastoderm, usually occurring around the animal pole region (Supplementary Figure 4). From two independent batches, about 12% (*n* = 65) of MZ*syne2b* embryos from young homozygous female parents displayed most severely delayed epiboly, with multiple disintegrated regions in the blastoderm before the end of gastrulation. This variation may result from the differential expressivity and the maternal age of mutant fish. There is also a possibility that other nesprins with similar functions may compensate for the loss of Syne2b. Indeed, expression of the dominant negative Syne1a KASH in MZ*syne2b* embryos further delayed epiboly (Supplementary Figure 5). Together, these analyses suggest that interference with maternal Syne2b function delays epiboly initiation and progression.

### Disrupted Cytoskeletal Organization in MZ*syne2b* Mutants

Analysis of the expression pattern of dorsal mesoderm markers *chordin* and *goosecoid* and the pan-mesoderm marker *tbxta* indicated that mesoderm patterning was not affected in MZ*syne2b* embryos. Nevertheless, the expression domain of *chordin* and *goosecoid* at shield stage became expanded laterally, while that of *tbxta* at 9 hpf was reduced along the anteroposterior axis, suggesting delayed convergence of lateral cells toward the embryonic shield at early stages of epiboly and reduced extension of axial mesoderm during late stages of gastrulation (Supplementary Figure 6). Given the binding activity of Syne2b to actin and the requirement of cytoskeletal dynamics for epiboly movement, we examined F-actin organization and YSN behaviors in stage-matched embryos (*n* = 6 from three independent batches for each condition).

**FIGURE 4:**
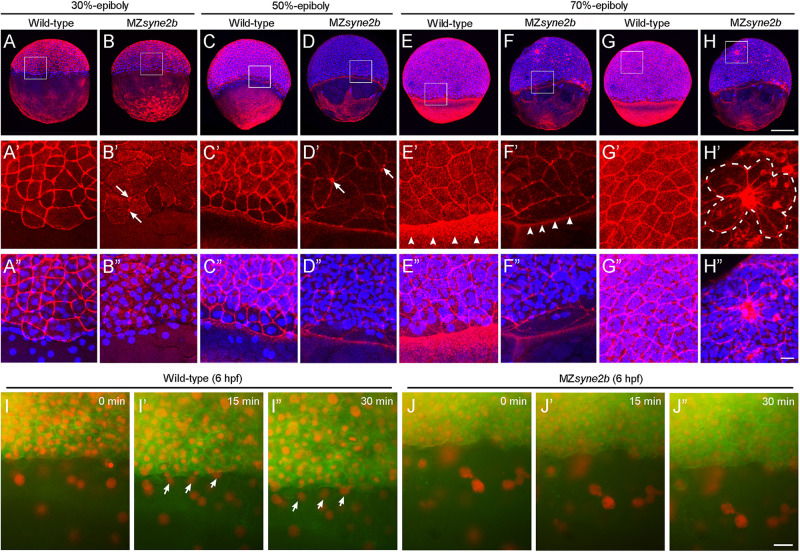
Disruption of Syne2b function affects F-actin organization, EVL cell shape and YSN movements. Phalloidin and DAPI staining of stage-matched embryos. **(A–A″)** Wild-type embryos at 30% epiboly show strong phalloidin staining in the cortex of EVL cells. A ring of YSN are apparent at the EVL margin. **(B–B″)** MZ*syne2b* embryos at 30% epiboly show reduced and clustered (arrows) phalloidin staining in EVL cells. Few YSN are present at the EVL margin. **(C–C″)** In wild-type embryos at 50% epiboly, phalloidin staining is present strongly in the cortex of EVL cells and uniformly in the yolk cell. YSN are still present at the EVL margin. **(D–D″)** In MZ*syne2b* embryos at 50% epiboly, phalloidin staining is clustered at multiple cell contact regions in the blastoderm (arrows) and is disrupted in the yolk cell. Few YSN are scattered in the yolk cell. **(E–E″)** In wild-type embryos at 70% epiboly, thick actin rings are formed around the blastoderm margin (arrowheads). **(F–F″)** MZ*syne2b* embryos at 70% epiboly form weak and thin marginal actin rings (arrowheads). YSN remain scattered in the yolk cell. **(G–G″)** Regular cortical F-actin and cell shape in the blastoderm of wild-type embryos. **(H–H″)** Severely disrupted cell shape and rearrangements in the blastoderm of MZ*syne2b* embryos, with the occurrence of rosette structures (broken lines). **(I,J″)** Still frames from time-lapse imaging show YSN movements at 50% epiboly. Note that YSN emerge from the front of the EVL margin during epiboly in wild-type embryos (arrows). Scale bars: **(A–H)** 200 μm; **(A′–H″)** 20 μm; **(I–J″)** 100 μm.

At 30% epiboly, strong and regular cortical F-actin, as revealed by phalloidin staining, could be observed in the cortex of EVL cells in wild-type embryos (Figures 4A–A″). However, weak and disrupted cortical F-actin was present in MZ*syne2b* embryos (Figures 4B–B″). Particularly, F-actin was concentrated at multiple cell contact regions (arrows in Figure 4B′). By this stage, DAPI-stained YSN were present ahead of the blastoderm margin in wild-type embryos (Figures 4A,A″), whereas they were rarely observed in MZ*syne2b* embryos (Figures 4B,B″). At 50% epiboly, besides the defective localization of cortical F-actin belt in EVL cells, F-actin bundles in the yolk cell was also disorganized in MZ*syne2b* embryos (Figures 4D–E″). The abnormal vegetal migration of YSN was evident, as further demonstrated by time-lapse imaging at 50% epiboly (Figures 4I–J″). This defect may be also correlated with a disorganization of microtubule arrays in the yolk cell (Supplementary Figure 7). At 70% epiboly when wild-type embryos formed a thick marginal actin ring (Figures 4E–E″), MZ*syne2b* embryos displayed a generalized F-actin disorganization, with strongly reduced F-actin in front of the EVL margin (Figures 4F–F″). Moreover, EVL cells in wild-type embryos displayed uniform cortical F-actin belt and took a polygonal shape (Figures 4G–G″), whereas F-actin became further concentrated at multiple cell contact regions in the blastoderm of MZ*syne2b* embryos (Figures 4H–H″). Strikingly, this caused the appearance of “actin-rich plaques,” which appeared to attach surrounding EVL cells, forming multiple pinwheel-like or rosette structures (Figures 4H′,H″ and Supplementary Figure 8). These defects could lead to reduced cellular cohesion, because blastoderm disintegration likely occurred at these regions in most severely affected MZ*syne2b* embryos (Supplementary Figure 8). The severe disruption of cortical F-actin associated with abnormal EVL cell shape changes and loss of epithelial integrity in MZ*syne2b* embryos was further confirmed by time-lapse recording at 70% epiboly using LifeAct-GFP (Supplementary Movies 1, 2). From mid-gastrula stage onward, some YSN recede from the marginal zone and converge toward dorsal and anterior regions (D’Amico and Cooper, 2001), thus no YSN could be observed in front of the EVL margin in wild-type embryos. However, scattered YSN were still present in the yolk cell of MZ*syne2b* embryos (Figures 4E,F and Supplementary Figures 8A′,B′), indicating abnormal dorsal convergence and anterior migration. Altogether, our results suggest that loss of maternal Syne2b function disrupts F-actin organization across the embryo, resulting in reduced epithelial integrity in the blastoderm and impaired YSN movements. All these defects contribute to delayed epiboly initiation and progression.

## Discussion

We have created *syne2b* gene mutation that should disrupt the nuclear localization KASH domain and nuclear-cytoskeletal connections. MZ*syne2b* embryos exhibited delayed epiboly initiation and progression, resulting in reduced elongation of the anteroposterior axis. Mechanistically, loss of maternal Syne2b function caused F-actin disorganization across the embryo. Particularly, abnormal accumulation of F-actin at multiple cell contact regions led to aberrant cell shape changes and impaired epithelial integrity. Moreover, YSN also showed defective migration during epiboly. Thus, our results demonstrate an important role for Syne2b in cytoskeletal organization during morphogenetic movements.

We showed that maternal *syne2b* transcripts are highly expressed in the blastodisc of cleavage stage embryos. During gastrulation, the expression of *syne2b* in the blastoderm and in the dorsal and ventral regions is consistent with a function in epiboly (Kudoh et al., 2001). Indeed, all MZ*syne2b* mutants displayed epiboly defects. A small proportion of embryos, generally derived from young female parents, also showed blastoderm disintegration at late stages of gastrulation. There is a possibility that variations or changes in phenotype expressivity with maternal age may result from genetic compensation by paralogous genes (El-Brolosy and Stainier, 2017). Syne1 and Syne3 are also anchored to the nuclear membrane and display similar functions as Syne2 (Ketema et al., 2007; Postel et al., 2011). Thus, they may have redundant activity as Syne2b in epiboly. Consistently, expression of the dominant negative Syne1a KASH in MZ*syne2b* embryos enhanced epiboly delay. A decline in the severity of embryonic phenotypes with maternal age has been also observed in other situations, such as *ichabod* mutants with defective dorsoanterior development (Kelly et al., 2000).

Interference with Syne2b function severely affected F-actin localization across the embryo, including disrupted cortical F-actin in EVL cells, reduced marginal F-actin ring, and disorganized F-actin bundles in the yolk cell. Most significantly, F-actin in EVL cells of MZ*syne2b* mutants was progressively accumulated at multiple cell contact regions. These “actin-rich plaques” likely caused abnormal local constriction of surrounding EVL cells, resulting in the formation of rosette structures. Thus, the disrupted cell arrangements impaired epithelial integrity and caused disintegration of the blastoderm during epiboly. Deletion of the C-terminal KASH domain likely disrupts Syne2b function to link different subcellular compartments, but further analyses are needed to examine the subcellular localization of the truncated protein. Given the binding activity of nesprins to actin, it is conceivable that perturbation of Syne2b function and localization should disrupt the organization of actin cytoskeleton, and as a consequence, cause abnormal cell shape changes. This is consistent with the intracellular scaffolding functions of nesprins in maintaining cellular architecture (Rajgor et al., 2012). Our *in vivo* observations are supported by previous studies showing that knockdown of Nesprin-1 and Nesperin-2 in human umbilical vein endothelial cells affects F-actin distribution and cell shape (King et al., 2014).

Removal of the KASH domain in nesprins also affects nuclear positioning and migration in mice (Zhang et al., 2007). Although it is unclear whether nuclear behaviors in EVL cells were also affected in MZ*syne2b* embryos, defective movements of YSN were apparent during epiboly. Through interaction with cytoskeleton, YSN pull the EVL toward the vegetal pole to cover the yolk cell during gastrulation (Kimmel et al., 1995; Li et al., 2017). Syne2b/nesprin-2 has been shown to regulate nuclear migration during retina development in mice (Yu et al., 2011). Interestingly, YSN in front of the EVL margin were absent or abnormally clustered in MZ*syne2b* embryos, suggesting impaired migration. It is likely caused by a disrupted cytoskeleton-nuclear membrane anchor activity. Besides F-actin bundles, microtubule arrays formed in the YSL and in the yolk cell are critical for driving the vegetal migration of YSN (Solnica-Krezel and Driever, 1994). Thus, disruption of microtubule networks in the yolk cell of MZ*syne2b* mutants may also contribute to impaired YSN movements during epiboly. This observation is consistent with a previous report showing that overexpression of the dominant negative Syne2a KASH slows migration speeds of YSN (Fei et al., 2019). Thus, our results further illustrate an important role of the LINC complex in coordinating nuclear movements.

In summary, we demonstrate for the first time that zebrafish maternal Syne2b is required for epithelial integrity and nuclear migration through regulation of cytoskeleton during morphogenetic movements. This study provides insights into different cytoplasmic and nuclear roles of Syne2b during early development.

## Data Availability Statement

The original contributions presented in the study are included in the article/Supplementary Material, further inquiries can be directed to the corresponding authors.

## Ethics Statement

The animal study was reviewed and approved by the Ethics Committee for Animal Research of Life Science of Shandong University.

## Author Contributions

Y-LL, X-NC, and TL performed the experiments, data collections and analyses. MS and D-LS designed and managed this study. D-LS wrote the manuscript. All authors approved the submitted version.

## Conflict of Interest

The authors declare that the research was conducted in the absence of any commercial or financial relationships that could be construed as a potential conflict of interest.
