# Supplementary material for: Syne2b/Nesprin-2 Is Required for Actin Organization and Epithelial Integrity During Epiboly Movement in Zebrafish
Source: Front Cell Dev Biol. 2021 Jun 17;9:671887. doi: 10.3389/fcell.2021.671887 (PMC8248263; doi:10.3389/fcell.2021.671887)
Supplement: Supplementary file 1 [file Data_Sheet_1.PDF]

Syne2b/nesprin-2 is required for actin organization and epithelial integrity during epiboly movement in zebrafish

Yu-Long Li[1#], Xiao-Ning Cheng[2#], Tong Lu[1#], Ming Shao[1*] and De-Li Shi[3,4*]

[1]School of Life Sciences, Shandong University, Qingdao 266237, China;

[2]Central People's Hospital of Zhanjiang, Zhanjiang 524045, China;

[3]Affiliated Hospital of Guangdong Medical University, Zhanjiang 524001, China;

[4]Laboratory of Developmental Biology, CNRS-UMR7622, Institut de Biologie Paris-Seine, Sorbonne University, 75005 Paris, France

[#]Contributed equally

[*]Corresponding authors:

shaoming@sdu.edu.cn (Ming Shao); de-li.shi@upmc.fr (De-Li Shi)

This PDF file includes:

Supplementary Table 1

Supplementary Figures 1-8

Supplementary Movies 1 and 2 captions

Supplementary Table 1. PCR primers used in this study.

Primer name	Sequence	Usage
dnsyne1a-F<br>*dnsyne1a*-R	5′-CAAGGTGGCGCTGCTGTGGTT-3′<br>5′-TCATACAGGAGGTGGGCCATTG-3′	Generation of dominant negative Syne1a KASH
syne2b-F<br>*syne2b*-R	5′-ATTCAGGAGATCTGGCACTTCAG-3′<br>5′-GATGTGGCATTATTATTCTC-3′	Generation of probe for in situ hybridization
goosecoid-F<br>*goosecoid*-R	5′-TCTCCAGTCCCACATCAA-3′<br>5′-GTATCGTCCTGCCATCGT-3′	Generation of probe for in situ hybridization
chordin-F<br>*chordin*-R	5′-TGGATTCTGCTGTCCGTTA-3′<br>5′-CTCCTCCTTCCTGTTGTCA-3′	Generation of probe for in situ hybridization
tbxta-F<br>*tbxta*-F	5′-ATCCCAGCCATTACTCCC-3′<br>5′-ATCTCCAACGCCAACCTC-3′	Generation of probe for in situ hybridization
syne1a-F<br>*syne1a*-R	5′-AGTGATTCCTGAGAGCCCTG-3′ (24997-25016)<br>5′-TCCATTAGACGCATGTAGCCC-3′ (25159-25179)	qRT-PCR analysis
syne1b-F<br>*syne1b*-R	5′-TCTCTCTATCCCGGCGCTAC-3′ (1206-1225)<br>5′-CGAAAGCTCTTAAAGGCCTGA-3′ (1332-1352)	qRT-PCR analysis
syne2a-F<br>*syne2a*-R	5′-CGGACAGCCTTATTGGGGAA-3′ (129-148)<br>5′-AATGTCCAGTAGAACGCCCC-3′ (221-240)	qRT-PCR analysis
syne2b-F<br>*syne2b*-R	5′-CAGAGTCGCCCGATTCCAC-3′ (28060-28078)<br>5′-GCTCCAAAGCAGGCGGTAA-3′ (28256-28274)	qRT-PCR analysis
syne3-F<br>*syne3*-R	5′-GACGTCCTGCTGATACGGAG-3′ (2882-2901)<br>5′-CCTGCACGCAAATCCTTGAG-3′ (3016-3035)	qRT-PCR analysis
GAPDH-F<br>*GAPDH*-R	5′-TTGCCGTTCATCCATCTTTG-3′<br>5′-TGCTGTAACCGAACTCATTGTC-3′	qRT-PCR analysis

NCBI reference sequences: *syne1a* (XM_021468628.1); *syne1b* (XM_009292831.2); *syne2a* (NM_001365331.1); *syne2b* (XM_021480883.1); *syne3* (NM_001083014.2). Nucleotide positions of PCR primer sequences are indicated after the primer sequences.

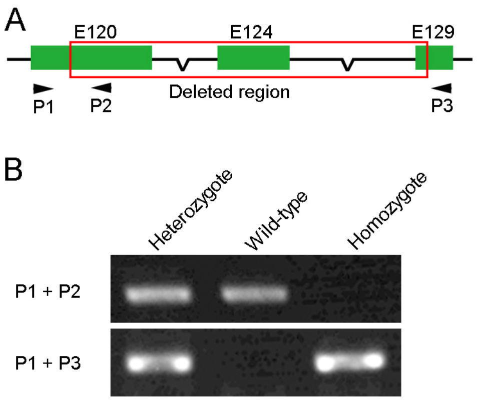

Supplementary Figure 1. PCR-based genotyping of *syne2b* mutants. **(A)** Schematic shows the position of PCR primers (arrowheads) used for amplification of genomic DNA. **(B)** PCR products obtained in wild-type embryos, heterozygous and homozygous *syne2b* mutants using different combinations of PCR primers (P1: 5'-GAGCGTGAGTTCTCGGACAG-3'; P2: 5'-ATTGTCAGAGATCAGTCAGTAA-3'; P3: 5'-TGACACTAAGAATAAGAGGG-3').

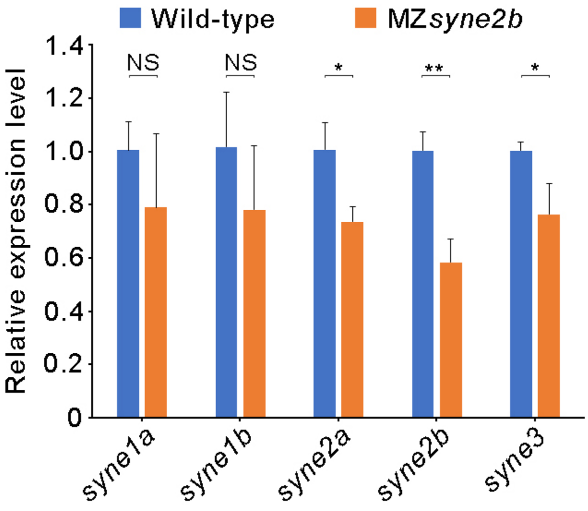

Supplementary Figure 2. Expression levels of different *syne* genes in MZ*syne2b* embryos. The expression of indicated genes in wild-type and MZ*syne2b* embryos was analyzed by qRT-PCR at 10 hpf. For each gene, the expression level in wild-type embryos was normalized to 1 as a reference. Bars represent the mean values ± S.D. from three independent experiments (NS, not significant; *, $p < 0.05$; **, $p < 0.01$).

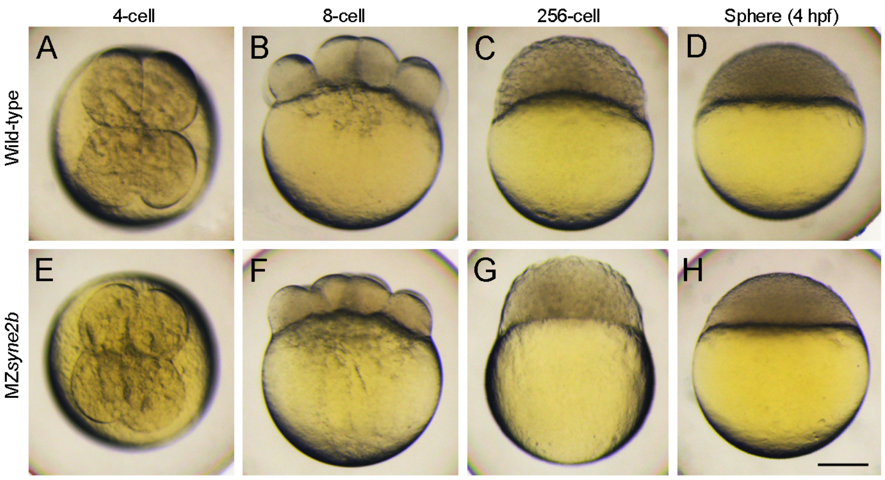

Supplementary Figure 3. Phenotypes of time-matched wild-type and MZ*syne2b* embryos before epiboly initiation. **(A-D)** Wild-type embryos at indicated stages. **(E-H)** MZ*syne2b* embryos undergo normal cleavage but are slightly higher at 256-cell stage. Scale bar: **(A-H)** 200 µm.

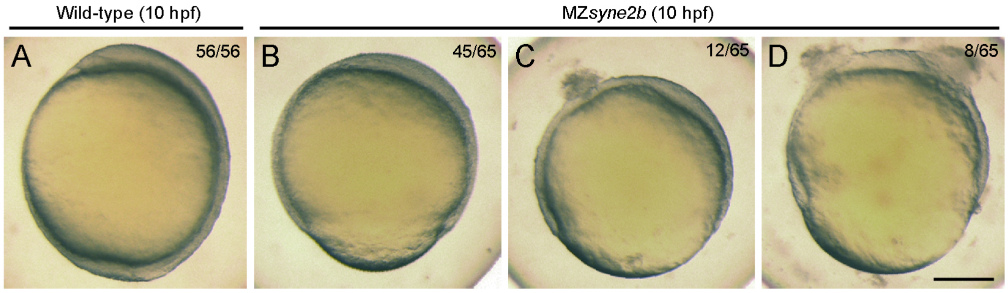

Supplementary Figure 4. Different degrees of epiboly delay and blastoderm integrity defects in MZ*syne2b* embryos. Data were scored from two independent batches (*n* = 65). **(A)** Wild-type embryos at 10 hpf complete epibloy (*n* = 56). **(B)** A majority of MZ*syne2b* embryos show epibloy delay at 10 hpf (69.2%, *n* = 65). **(C)** More severely affected MZ*syne2b* mutant embryos with further delayed epiboly (18.5%, *n* = 65). Blastoderm disintegration occurs in one region. **(D)** Most severely affected MZ*syne2b* mutant embryos with strongly delayed epiboly and blastoderm disintegration at multiple regions (12.3%, *n* = 65). Scale bar: **(A-D)** 200 µm.

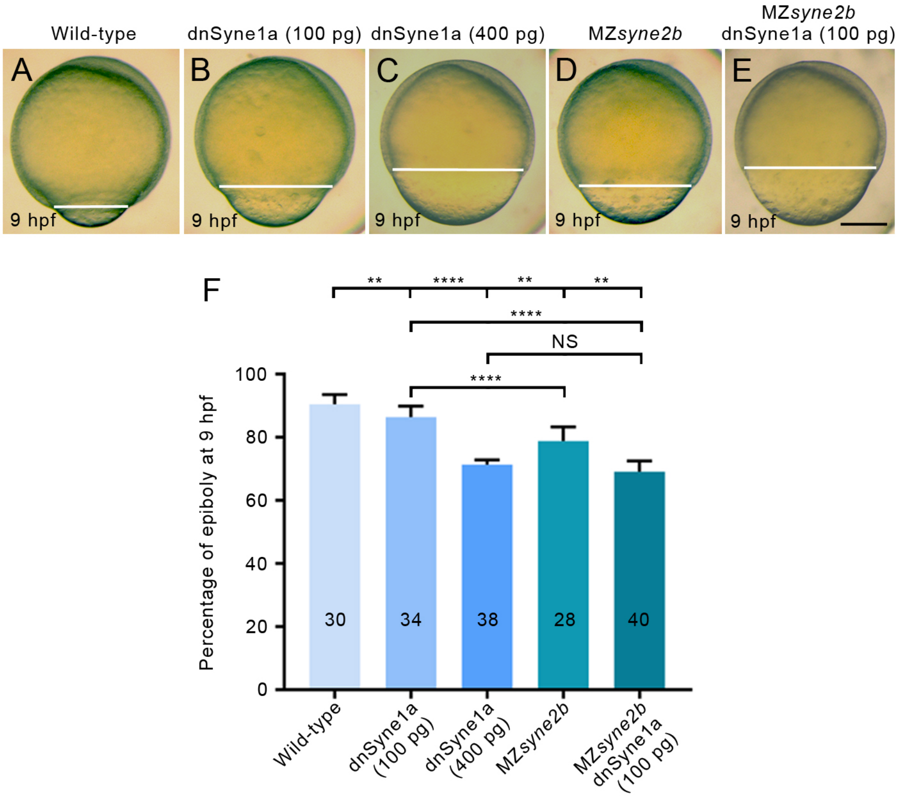

Supplementary Figure 5. Expression of the dominant negative Syne1a KASH in MZ*syne2b* embryos enhances epiboly delay. Horizontal lines indicate epiboly progression. **(A)** Wild-type embryos at 9 hpf reach 90% epiboly. **(B,C)** Expression of the dominant negative Syne1a KASH in wild-type embryos causes epiboly delay in a dose-dependent manner. **(D)** MZ*syne2b* embryos at 9 hpf show delayed epiboly. **(E)** MZ*syne2b* embryos injected with a low dose of *dnSyne1a* mRNA show further delayed epiboly. **(F)** Statistical analyses of epiboly progression at 9 hpf. Numbers in the columns indicate total embryos used for statistical analyses. Bars represent the mean values ± S.D. from two independent experiments (NS, not significant; **, $p < 0.01$; ****, $p < 0.0001$). Scale bar: **(A-E)** 200 µm.

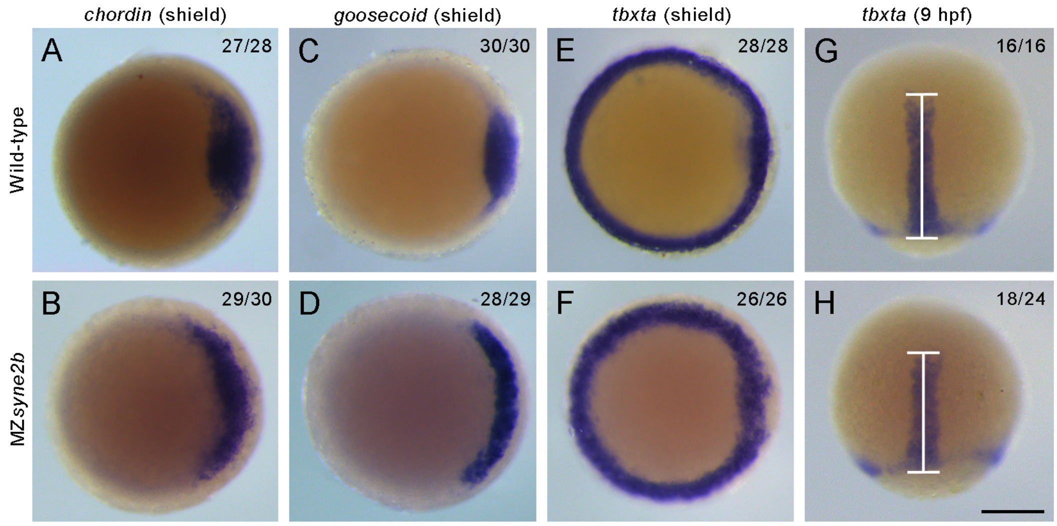

Supplementary Figure 6. Embryonic patterning is not affected in MZ*syne2b* embryos. In situ hybridization analysis of the expression pattern of dorsal mesoderm and pan-mesoderm genes in wild-type and MZ*syne2b* embryos. **(A-F)** Expression of *chordin*, *goosecoid* and *tbxta* at shield stage. Dorsal is to the right. **(G,H)** Expression pattern of *tbxta* in the notochord at 9 hpf. Dorsal view with anterior up. The expression domains of *chordin* and *goosecoid* at shield stage are wider mediolaterally, indicative of delayed convergence of lateral cells toward the embryonic shield, while the expression pattern of *tbxta* at 9 hpf is reduced along the anteroposterior axis, suggesting impaired extension of midline cells. Statistical numbers are indicted in the upper right corner. Scale bar: **(A-H)** 200 μm.

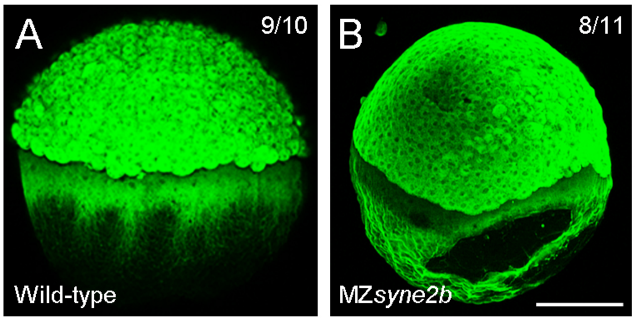

Supplementary Figure 7. Disorganization of microtubules in the yolk cell of MZ*syne2b* embryos. Immunofluorescence staining of ß-tubulin at 50% epiboly. **(A)** Microtubule arrays in wild-type embryos. **(B)** Disrupted microtubule arrays in the yolk cell of MZ*syne2b* embryos. Statistical numbers are indicted in the upper right corner. Scale bar: **(A,B)** 200 μm.

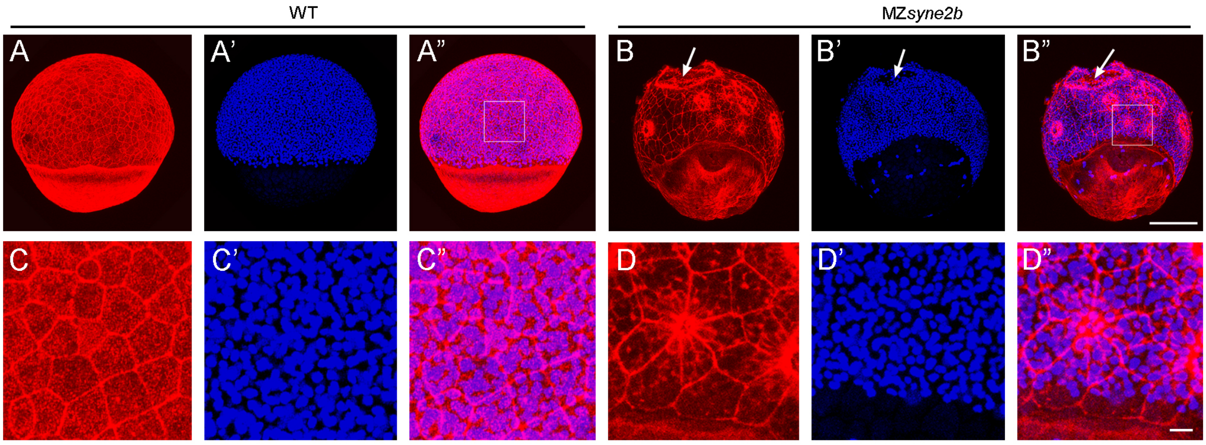

Supplementary Figure 8. F-actin organization and epithelial integrity in severely affected MZ*syne2b* embryos. **(A-A”)** Phalloidin and DAPI staining of wild-type embryos at 70% epiboly stage. Note that YSN are no longer present in front of the EVL margin at this stage. **(B-B”)** Phalloidin and DAPI staining of stage-matched MZ*syne2b* embryos, showing strongly disorganized F-actin associated with disrupted cell shape in the blastoderm. Scattered YSN are present in the yolk cell. Arrow indicates a disintegrated blastoderm region. **(C-C”)** Higher magnification shows uniform phalloidin staining and regular cell shape in the blastoderm of wild-type embryos. **(D-D”)** Higher magnification shows abnormal accumulation of F-actin at multiple cell contact regions and the presence of rosette structures in the EVL of MZ*syne2b* embryos. Scale bars: **(A-B”)** 200 µm; **(C-D”)** 20 µm.

Supplementary Movie 1. Cell shape changes in the EVL during epiboly in wild-type embryos. Live time-lapse imaging of LifeAct-GFP was performed at 70% epiboly.

Supplementary Movie 2. Cell shape changes in the EVL during epiboly in MZ*syne2b* embryos. Live time-lapse imaging of LifeAct-GFP was performed at 70% epiboly. Arrows point “actin-rich plaques”; thick arrow indicates cell detachment.
